# Parental mosaicism in Marfan and Ehlers–Danlos syndromes and related disorders

**DOI:** 10.1038/s41431-020-00797-3

**Published:** 2021-01-07

**Authors:** Bertrand Chesneau, Aurélie Plancke, Guillaume Rolland, Nicolas Chassaing, Christine Coubes, Elise Brischoux-Boucher, Thomas Edouard, Yves Dulac, Marion Aubert-Mucca, Thierry Lavabre-Bertrand, Julie Plaisancié, Philippe Khau Van Kien

**Affiliations:** 1 UF de Génétique Médicale et Cytogénétique, Centre Hospitalier Universitaire de Nîmes, Nîmes, France; 2 Centre de Référence du Syndrome de Marfan et des Syndromes Apparentés, Hôpital des Enfants, Centre Hospitalier Universitaire de Toulouse, Toulouse, France; 3 Service de Génétique Médicale, Hôpital Purpan, Centre Hospitalier Universitaire de Toulouse, Toulouse, France; 4 Service de Génétique Clinique, Département de Génétique Médicale, Maladies Rares et Médecine Personnalisée, Hôpital Arnaud de Villeneuve, Centre Hospitalier Universitaire de Montpellier, Montpellier, France; 5 Centre de Génétique Humaine, Centre Hospitalier Universitaire de Besançon, Besançon, France

**Keywords:** Aortic diseases, Medical genetics, Disease genetics, Genetic counselling

## Abstract

Marfan syndrome (MFS) is a heritable connective tissue disorder (HCTD) caused by pathogenic variants in *FBN1* that frequently occur de novo. Although individuals with somatogonadal mosaicisms have been reported with respect to MFS and other HCTD, the overall frequency of parental mosaicism in this pathology is unknown. In an attempt to estimate this frequency, we reviewed all the 333 patients with a disease-causing variant in *FBN1*. We then used direct sequencing, combined with High Resolution Melting Analysis, to detect mosaicism in their parents, complemented by NGS when a mosaicism was objectivized. We found that (1) the number of apparently de novo events is much higher than the classically admitted number (around 50% of patients and not 25% as expected for *FBN1*) and (2) around 5% of the *FBN1* disease-causing variants were not actually de novo as anticipated, but inherited in a context of somatogonadal mosaicisms revealed in parents from three families. High Resolution Melting Analysis and NGS were more efficient at detecting and evaluating the level of mosaicism compared to direct Sanger sequencing. We also investigated individuals with a causal variant in another gene identified through our “aortic diseases genes” NGS panel and report, for the first time, on an individual with a somatogonadal mosaicism in *COL5A1*. Our study shows that parental mosaicism is not that rare in Marfan syndrome and should be investigated with appropriate methods given its implications in patient’s management.

## Introduction

Marfan syndrome (MFS, [OMIM #154700]) is caused by heterozygous variants in *FBN1*, which alter the function of the encoded fibrillin 1 protein, a component of the extracellular microfibrils [1]. This heritable connective tissue disorder (HCTD) is dominantly inherited and characterized by high penetrance and variable expressivity with some patients displaying only discrete signs and some with early-onset MFS [2]. The broad phenotypic expression includes manifestations mainly in the cardiovascular (i.e., aortic root aneurysm), ophthalmologic (i.e., *ectopia lentis*), and skeletal systems (i.e., arachnodactyly, scoliosis, *pectus* deformity). Diagnosis is established following the Revised Ghent Nosology [1].

The frequency of de novo MFS-affected individuals is classically reported to be around 25% [3]. True de novo individuals (disease-causing variant in only one gamete) are hard to distinguish from MFS sporadic individuals occurring from mosaicism. In this latter situation, there are two levels of possible mosaicism [4]. On one hand, in the proband when a disease-causing variant appears during the early stages of the embryologic development (also named post-zygotic event) leading to somatic mosaicism. On the other hand, in one of the two parents, the disease-causing variant can appear during gametogenesis (germline or gonadal mosaicism) or, in the same way, during the early stages of the embryologic development affecting both soma and germ cells (somatogonadal mosaicism). This last mechanism, which has been previously reported in MFS [5–9] induces a risk of recurrence in siblings of an apparently sporadic proband [6]. Parental molecular analysis revealed the majority of reported individuals with post-zygotic mosaicisms in *FBN1*. Despite the fact that most patients with proven somatogonadal mosaic were asymptomatic or displayed discrete MFS signs [5, 8], some of them displayed aortic dilatation [6, 7].

Diagnosis of *FBN1* mosaicism is a challenge for the clinician dealing with patients harboring subtle signs of MFS. It is also challenging for the diagnostic laboratory due to the limits of detection sensitivity for a mosaic variant detection. Multiple technologies have been tested and developed in order to be more sensitive in mosaic variant detection [10–13].

Although somatogonadal mosaicism in MFS appears to be rare, its frequency is not known and has only been published in case reports. In particular, no systematic molecular parental studies have been performed in the way that was done in vascular Ehlers–Danlos Syndrome (vEDS) or in developmental disorders [14, 15] except as a communication at the 2020 ESGH meeting [16]. To try to estimate the frequency of de novo and parental mosaic variants in MFS, we reviewed all the individuals with a MFS confirmed by a disease-causing variant in *FBN1* in our center. We report here three novel parents with a somatogonadal mosaicism for the disease-causing in *FBN1* variant identified in their child. This allows us to estimate, to an accuracy of around 5%, the frequency of parental mosaicism in MFS.

Mosaicism has been reported in other genes of HCTD, in particular in Loeys–Dietz syndrome with mosaic variants in *TGFBR1* and *TGFBR2* [17, 18]. We therefore, reviewed all affected individuals for other HCTD genes but also familial thoracic aortic aneurysm/dissection (FTAAD) genes studied in our center. We found a parental mosaicism in *COL5A1*, an occurrence which has never before been reported to our knowledge.

## Materials and methods

### Patients

This study was designed in compliance with the tenets of the Helsinki Declaration. Informed consent was obtained from all individuals included in this study, which was approved by our local ethic committee (Nîmes University Hospital).

We included all the 333 probands with a (likely) pathogenic variant in *FBN1* referred for molecular diagnosis in our center during the past 15 years (2004–2019) in order to determine the frequency of mosaicism in this disorder. The diagnosis was performed initially by direct sequencing (of *FBN1*, *TGFBR1*, and *TGFBR2* from 2004 to 2013) and by NGS (detailed below and in Supplementary Material 1).

We also collected data of patients with HCTD harboring a disease-causing variant in a gene other than *FBN1* (genes list in Supplementary Material 1). However, because of the low number of patients for each of these genes, they were not included in our statistical analyses.

Clinical data and family history were obtained through a questionnaire completed by the medical referent. Probands with neither data on their family history nor molecular parental studies were excluded. The condition was considered as “familial” in presence of another affected relative and as “sporadic” when family history was not suggestive. It should be noted, as the objective was to determine whether the variant was likely to be inherited or not, that the proband’s offspring was not taken into consideration. The variant was considered inherited if one of the parents or another relative (if parental transmission was suspected) was heterozygous for the variant.

We also reviewed all the siblings who benefited from molecular analysis after parental studies showing de novo occurrence, in order to look for a recurrence.

### Molecular testing of relatives

We routinely use two methods for molecular testing of relatives in our center: direct sequencing and High Resolution Melting Analysis (HRMA). It should be noted that the analysis of six microsatellites markers in the *FBN1* locus is systematically performed to confirm the paternity and maternity as well as DNA contamination.

Direct sequencing was performed for both parents of 163 probands (141 with disease-causing variant in *FBN1*, 22 in another FTAAD gene). We performed targeted PCR amplification using specific primers on the proband’s and his/her relatives’ genomic DNA extracted from blood and cheek cells (when available). PCR products were then sequenced using an ABI3130XL or an ABI3500XL (Applied Biosystems, Foster City, California, U.S.A.). DNA sequence variants were identified using SeqPilot software (JSI medical systems, Ettenheim, Germany).

HRMA, which was developed secondarily in our lab (2012), was performed when applicable in the parents of 81 out of 163 probands (62 with disease-causing variant in *FBN1*, 19 in another FTAAD gene). This second method was developed as a complement to direct sequencing because of its better sensitivity, in particular for mosaic variants detection although it has some limitations (i.e., single nucleotide polymorphism near the variant) [11]. We performed targeted real-time PCR with specific primers (different couples than those used for direct sequencing) using Light Cycler 480 (Thermo Fisher scientific, Waltham, Massachusetts, USA). HRMA was also used for semi-quantitative analysis in mosaic patient as described elsewhere [11]; to estimate the mosaicism using HRMA, we performed a dilution range of the familial variant in a non-mutated DNA.

When we detected a somatogonadal mosaic variant by either HRMA, direct sequencing or both in samples of parents, we then performed a NGS analysis in order to have a quantitative estimation of the mosaicism level and a comparison between these different methods. The NGS FTAAD panel used includes 35 genes (list in Supplementary Material 1). This also allowed checking for DNA contamination. Four parents were identified: three with a mosaic variant in *FBN1* (M0078, M0277, M0336) and one with a mosaic variant in *COL5A1* (M1144). These novel *FBN1* and *COL5A1* variants reported here have been deposited for public access in the Global Variome shared LOVD at www.lovd.nl/FBN1 and www.lovd.nl/COL5A1 with individual IDs #00306166 to #00306169.

Library preparation was performed using 3 µg of DNA. Capture of all the coding exons and their flanking intronic sequences were performed using Sureselect technology (Agilent, Santa Clara, California, USA). All the samples were run on the Ion chef & S5™ system (Thermo Fisher Scientific). Run parameters and data output from each run were obtained and compared against specifications outlined by the manufacturer. Bioinformatics data analysis steps were performed using NextGENe software (Softgenetics, State College, Pennsylvania, USA) and Alamut Visual (Integrative Biosoftware, Rouen, France).

## Results

### Estimation of the de novo variant frequency and parental mosaicism in MFS

**Fig. 1:**
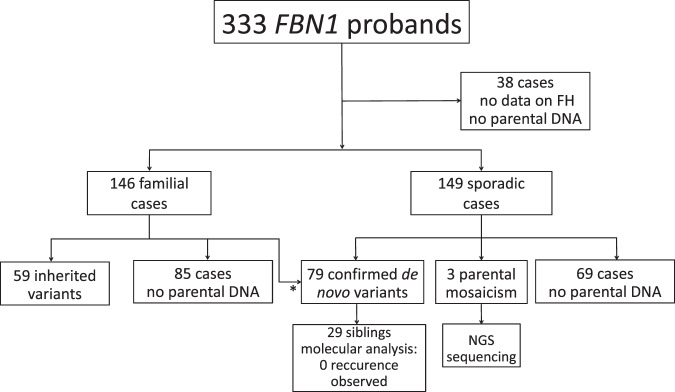
Flow chart of the study. FH family history, NGS next-generation sequencing. * Two cases reclassified as de novo after parental molecular study.

We identified 333 pedigrees with a disease-causing variant in *FBN1*. For 38 out 333, we had no information on family history with no available parental DNA and thus, they were excluded from our study (Fig. 1). Among the 295 patients included, half had a family history evocative of MFS (146/295) and half were sporadic (149/295). In 141 probands, molecular analysis (direct sequencing combined with HRMA for 62 probands) was performed in both parents. The variant was inherited from a heterozygous parent in 59 of them (41.8%) and appeared de novo in 79 probands (56.0%). In three pedigrees (MF0014, MF0050, MF0140), analyses revealed a mosaicism for the variant in a parent (2.1%). When taking into account only the analyses made by both HRMA and direct sequencing, somatogonadal mosaicism in one parent was observed at a frequency of 4.8% (3/62; 1.1–13.6% for a 95% CI). In addition, when the variant was inherited, we tried to extend the study of the familial variant (when DNA was available) to earlier generations. We did not detect any other affected individuals with mosaicism (data not shown). It should be noted that the absence of DNA contamination was checked by NGS analysis and by the use of six microsatellites at the *FBN1* locus.

In two families (MF020, MF272), despite a familial history suggestive of MFS, the *FBN1* disease-causing variant occurred de novo (NM_000138.5: c.2948C>T and c.5060G>A, respectively). In the MF020 family, the half-sister was reported with a mild mitral insufficiency, arachnodactyly (thumb without wrist sign), and joint hypermobility. In the MF272 family, the prescriber suspected a MFS in the sister, who displayed a slight *marfanoid habitus* (normal ophthalmologic examination and echocardiography). No other findings suggestive of MFS were reported in other relatives of the two families. Notably, parental echocardiography was normal. Both direct sequencing and HRMA confirmed that the variant was de novo and not found in the suspected affected sibling. Sample origins were checked by microsatellite analyses. This allowed the elimination of any clinical doubt, and no further molecular analysis was performed.

**Fig. 2:**
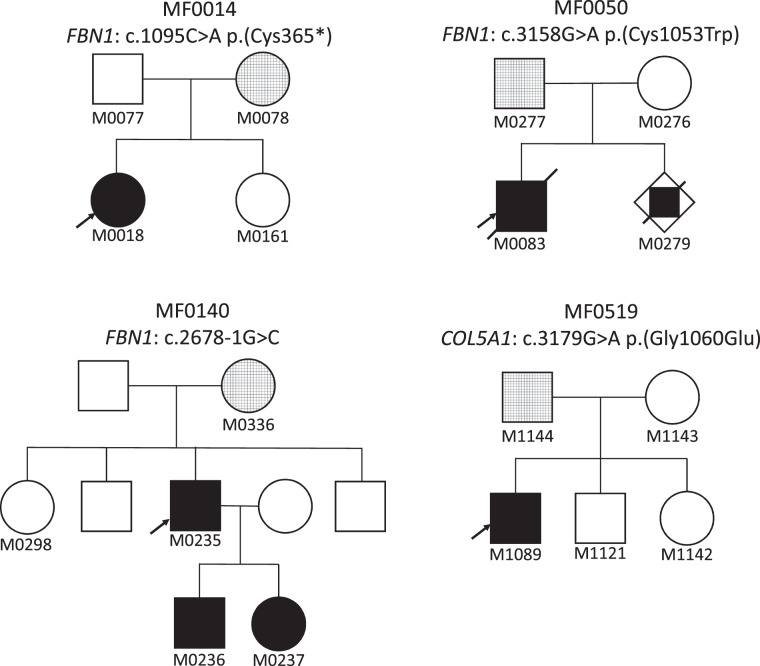
Pedigrees of the four families with parental mosaicism. Tested individuals are mentioned by their anonymous identification. Affected heterozygous patients are indicated with filled symbols. Patients with mosaicism are indicated by hatched symbols.

In three families (MF0014, MF0050, MF0140), parental segregation analysis revealed a somatogonadal mosaicism of the, respectively, *FBN1* (NM_000138.5): c.1095C>A; c.3158G>A and NG_008805.2(NM_000138.5):c.2678-1G>C variants in one parent (M0078, M0277, M0336). Clinical data of these pedigrees are summarized in Table 1 and Fig. 2.

**Table 1:** Clinical data of parents with somatogonadal/germline and somatic mosaicism in *FBN1* and their affected children.

Family	Patient	Age^a^ (years)	Cardiovascular	Ocular	Systemic features	Systemic score
MF0014	M0018	28	TAAD (surgery)	–	Scoliosis, PD, AraD, PP, CFD^b^	8
	M0078	55	–	–	Dolichostenomelia	1
MF0050	M0083	Birth	TAAD (+6 SD), severe MI, tricuspid valve prolapse	Myopia (high)	Scoliosis, PD, PP, AraD, joint laxity, distal arthrogryposis, CFD^b^	9
	M0279	In utero	ND	ND	AraD, arthrogryposis	ND
	M0277	38	TAAD (+2, 5 SD), BAV	ND	AraD	ND
MF0140	M0235	37	ND	ND	ND	ND
	M0336	65	–	Myopia (mild), LE: amblyopia, peripapillary atrophy	Scoliosis, AraD, skin striae	5

Normal examination, *AraD* arachnodactyly, *BAV* bicuspid aortic valve, *CFD* craniofacial dysmorphy, *LE* left eye, *MI* mitral insufficiency, *ND* not determined, *PP* *pes planus*, *PD* pectus deformity, *TAAD* thoracic aortic aneurysm or dissection.

^a^Age at diagnosis.

^b^Systemic score and craniofacial dysmorphy as evaluated in the revised Ghent nosology for the Marfan syndrome (Loeys et al. [1]).

**Fig. 3:**
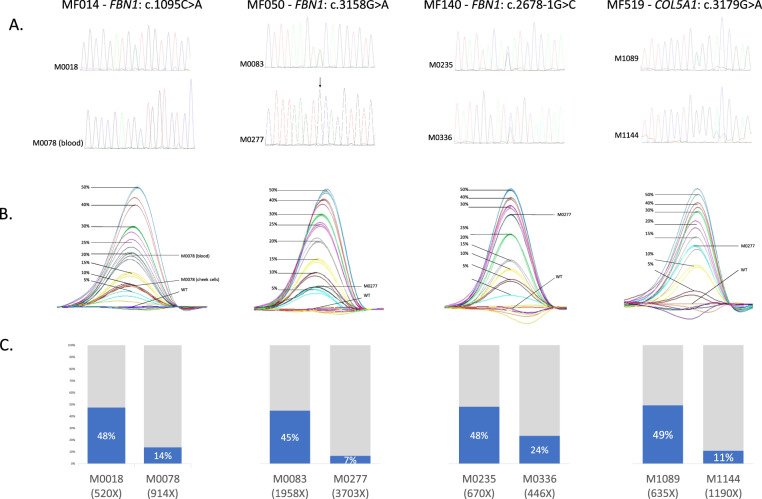
Molecular analyses of the probands and in their parents with a mosaic variant. **A** Electrophoregrams of direct sequencing showing the causal variant present at heterozygous state in the proband and in mosaic in the parent’s blood. Note the absence of visualization of the variant in family MF050 (as indicated by arrow). **B** Melting curves of High Resolution Melting Analysis showing the dilution series for variants in *FBN1* or *COL5A1* for each family, with the superposition of the profile of mosaic patients. WT wild type. **C** Variant allele fraction (VAF) in blood obtained by next-generation sequencing showing the level of mosaicism (depth at the position is specified below patient number).

In MF0014, a sample of cheek cells was available for the parent M0078. HRMA revealed a lower level of mosaicism in the cheek cells (variant allele frequency (VAF): 10%) than in blood cells (VAF: 20%) for the disease-causing variant c.1095C>A in *FBN1* (Fig. 3B).

In MF0050, we confirmed a recurrence of an early-onset MFS. The proband in question (M0083) was initially suspected to have Beals syndrome [MIM#121050] at birth because of congenital arthrogryposis, suggestive craniofacial, and marfanoid features. MFS was later suspected because of the development of an aortic aneurysm during the first year (Valsalva sinus: 24 mm +5 Z-score, body surface area 0.42 m² at 1 year of age) and a high myopia. At the last evaluation at 11 years of age, the proband’s thoracic aortic aneurysm was stable with 37.5 mm at the Valsalva sinus (+6 Z-score, body surface area 0.9 m²) and he developed a severe mitral insufficiency as well as a progressive scoliosis (Cobb angle 48°). His brother (M0279) died in utero at 38 weeks of amenorrhea without a cause identified during the postmortem examination (hypotrophic placenta was noted). This examination also revealed craniofacial features (prefrontal edema, hypertelorism, small chin, low set hair in the forehead), club hands with *adductus* thumbs, arthrogryposis of elbows, hips, and knees. A molecular study confirmed the presence of the heterozygous c.3158G>A *FBN1* variant in his genomic DNA. The echocardiography of their father (M0277), who harbors the variant in the mosaic state (VAF: 7% in blood, Fig. 3), shows a mild aortic aneurysm at 41 mm (+2.5 Z-score, body surface area 2.0 m²).

### Mosaicism in other HCTD

We identified 51 families with a FTAAD caused by genes other than *FBN1*. The diagnosis was confirmed with the identification of a disease-causing variant in one of the following genes: *TGFBR2* (*N* = 21), *TGFBR1* (*n* = 8), *SMAD3* (*n* = 7), *TGFB3* (*n* = 4), *ACTA2* (*n* = 2), *FBN2* (*n* = 2), *LOX* (*n* = 2), *MYH11* (*n* = 1), *MYLK* (*n* = 1), *SKI* (*n* = 1), and *COL5A1* (*n* = 1). Among them, 22 molecular analyses of both parents were performed and we discovered a paternal mosaicism in *COL5A1* identified in one family (MF0519). We did not detect any other variant mosaicisms.

In family MF0519 (pedigree in Fig. 2), the proband was an 8-year-old boy (M1089) who displayed a classical EDS (cEDS) with skin hyperextensibility, atrophic scarring, *molluscoid* pseudotumors, and generalized joint hypermobility (Supplementary Material 2). Molecular analysis confirmed the diagnosis with a disease-causing *COL5A1* (NM_000093.5) variant: c.3179G>A. It was found at mosaic state in the 39 year-old father’s DNA (VAF:11% in blood, Fig. 3). Careful clinical examination did not find any skeletal, skin or other diagnostic criteria in the father. Both of his other children did not inherit the variant.

### Comparison of direct sequencing, HRMA, and NGS to detect a mosaic variant

HRMA and NGS were both able to detect the four mosaic variants. Direct sequencing failed to detect the *FBN1* c.3158G>A variant with the lowest level of mosaic (M277) (Fig. 3). Estimation of the VAF was concordant between HRMA and NGS (0–6% difference).

## Discussion

This study shows that the frequency of parental mosaicism in MFS is not that rare. We can estimate the parental mosaicism as around 4.8% (1.1–13.6%; 95% CI) with the retrospective analysis of data collected in our clinical laboratory. Interestingly, this estimation of MFS mosaicism is concordant with the conclusions of Yan et al. who reported, at the 2020 ESHG Meeting, a parental mosaicism of 3.6% in a population of 563 patients with suspected MFS or thoracic aortic aneurysm/dissection [16]. Until then, observations of mosaicism had only been reported through individual studies and were therefore considered as rare phenomena [5–9, 19–22]. However, this has an important impact for MFS for both the assessment of the recurrence risk in siblings and medical follow-up of mosaicism-affected individuals. The recurrence risk is indeed correlated to the timing of variant’s appearance: from high (50%) if the variant is constitutive in the parent, to null if the variant results from a post-zygotic event in the proband [4, 14]. Somatogonadal mosaicism in a parent confers an intermediate and undefined risk between 0 and 50%. Campbell et al. [23], modeled the multiple factors involved in estimating this recurrence risk (parent of origin and age for male, sexual dimorphisms during gametogenesis, mosaicism, sibling structure, etc.). They show that the detection of a somatic parental mosaicism in blood is the most important factor. It considerably increases the risk of recurrence, which can be estimated according to their calculations to 5.21% when a mosaic is detected in one parent (regardless of gender and age) in the case of a single affected child (the risk is modulated according to the number of children and their status). The recurrence risk is much lower when no mosaics are detected in the blood of the parents (depending on sex and age for fathers, 0.048–2.6% for a single affected child). This estimate of the recurrence risk although to be considered with caution undoubtedly explains why despite a significant frequency of mosaicism detected in our study, recurrence cases are rarely observed in clinical practice. Therefore, clinical evaluation of siblings as well as molecular testing should be systematic because there is always a risk that an apparently de novo situation hides a parental germline mosaicism. It is also an important risk assessment to make for prenatal and preimplantation genetic diagnosis considerations.

In a large majority of parents, only blood was analyzed and we cannot exclude the possibility of a gonadal mosaicism absent from blood. This phenomenon is frequent in other diseases and can be clinically relevant. For example, in Cornelia de Lange syndrome, disease-causing mosaic variants in *NIPBL* are detected in cheek cells but not in blood in 15–25% of patients [24]. In 1999, Rantamäki et al. reported an individual with germline mosaicism associated with a recurrence of MFS in two siblings [9]. The disease-causing *FBN1* variant was not detected in either of the parents despite molecular analysis of different tissues for each of them. Due to a lack of other samples, this comparison between different tissues was performed only for parent M0078 (cheek cells and blood). The *FBN1* variant was detected in both tissues with a lower level of mosaicism in cheek cells than in blood (Fig. 3B).

This study was retrospective and therefore the technologies used for the molecular analysis of parents varied, with only direct sequencing being used in some patients and direct sequencing combined with HRMA in others. Our results confirm the limits of direct sequencing to detect mosaic variants [11–13] as it failed to detect the variant in MF050 with the lowest estimated mosaic level (VAF: 5–10%, Fig. 3). Variants with a similar mosaic level could have been missed when analyzed only by direct sequencing. This underlines the importance of using the most-sensitive technique to detect such variants. HRMA was associated with direct sequencing in order to increase sensitivity in familial variant detection [11–13]. High deep NGS can detect variants with an allelic balance below 1% [4, 10] and thus offers a better sensitivity to detect mosaic variants than direct sequencing. Digital droplet PCR is also a more efficient method. The latter two methods are however not routinely applied in clinical practice to screen for mosaic variants during parental molecular analysis (due to cost and time constraints). We thus use HRMA, which has the advantage of being a simple low-cost, single-step, closed-tube method whenever possible as the second independent technique for the screening of relatives. Furthermore, NGS may help to solve rare highly suggestive MFS patients with negative molecular test results, which may be explained by an uncharacterized mosaic variant. Our routine bioinformatics pipelines are adjusted not to retain variants with an allelic ratio lower than 20, as variants beneath this threshold are difficult to differentiate from the background noise and their analyses are not compatible with routine molecular analyses. This artificial limit is removed when analyzing mosaic variants. Furthermore, we observed that low-grade mosaicism (VAF < 20%) is not expected to cause typical MFS.

Although there are no apparent correlation between the level of mosaicism and the clinical severity in the few patients described in the literature and herein with a variant mosaicism in *FBN1* [5–9, 19–22], clinical signs tend to be milder in patients with a mosaic variant than in heterozygous patients. Only one of our patients (with a VAF of 7%) displayed cardiovascular signs with a mild aortic root dilatation (patient M0277, Table 1). Another affected individual with mild aortic root dilatation was previously described (low level of mosaicism) [6] and one patient who made a type B aortic dissection [7]. Other cardiovascular anomalies were reported, in particular mild mitral and tricuspid insufficiency [6, 22]. Two patients had an eye examination (M0078, M0336) that did not reveal any anomaly beyond a mild myopia in one of them (M0336). Interestingly, Šípek et al. described one patient with an unilateral *ectopia lentis* related to a somatic mosaicism (VAF:10–25% in blood) [22]. The three MFS mosaic patients that we report displayed various systemic signs (i.e., arachnodactyly, scoliosis, *striae*) but the systemic score according to the revised Ghent’s nosology [1] remained negative (<7). Therefore, despite the milder clinical presentation of patients harboring a mosaic variant in *FBN1*, an appropriate follow-up is necessary to prevent complications, in particular aortic dissection. The question of whether a life-long betablockade therapy is indicated or not, independently of aortic dilatation, is unsolved. Furthermore, we recommend an echocardiography at least once during a parental study because of the residual risk of missing a mosaicism during the molecular study (notably by direct sequencing only) and in view of observations of aortic aneurysms/dissection with low levels of mosaicism (M0277) [6, 7]. On the contrary, in the absence of affected offspring, we can assume that this milder phenotype would result in undiagnosed patients.

One of our patients (M00277) displayed mosaicism for the *FBN1*:c.3158G>A [p.(Cys1053Tyr)] variant, located in the exons 24–32 region (formerly “neonatal region”) associated with early-onset MFS [25] accordingly to the severe phenotype noticed in his children. Such a situation was previously reported in two families [20, 22]. Our study is, however, the first to our knowledge to report a molecularly proven recurrence of early-onset MFS. In order to refine the estimation of the recurrence risk, other studies have analyzed sperm samples of mosaic fathers [26, 27]. Although it can help some couples to have this estimation, in most instances, it does not add valuable information. A lower allele fraction in a sperm sample than in a blood sample does not remove the recurrence risk.

Variants of various types have been found to be associated with parental mosaic events, most of them being single nucleotide variations or insertion/deletions [5–9, 19, 20, 22]. However, a complete *FBN1* deletion [21] has been also reported. We found parental transmission in both sexes: a total of six maternal mosaicisms and four paternal mosaicisms have been reported, including our new patients [5–8, 20–22]. Thus, there does not seem to be a unique mitotic mechanism resulting in parental mosaicism.

The frequency of de novo variants that we observed (47.0–50.0%) is higher than the classically admitted 25% of de novo variants [3] but similar percentages have been observed in a large international cohort of 1013 probands with MFS [2]. Faivre et al. discussed the important number of de novo mutations found in the probands of their study and raised the possibility that this higher proportion “does not reveal a higher-than-reported mutation rate but reflects practice and screening results from the contributing centers worldwilde” [2]. It is likely that large characteristic Marfan pedigrees have been first screened.

Suggestive family histories with de novo variants were observed twice in our patients. Reevaluation of the two suggestive affected relatives with the negative molecular analysis showed no major signs of MFS according to the revised Ghent nosology. In addition, in multiple families, molecular analyses revealed that the variant was inherited from one parent referred to as asymptomatic. Careful retro-phenotyping showed MFS clinical involvement in most of the latter studies. Thus, systematic molecular testing combined with careful clinical evaluation (including echocardiography) of all first-degree relatives is essential.

Previous studies report somatogonadal mosaicism in other HCTD, for example in Loeys–Dietz syndromes [17, 18] and vEDS [14]. We report here, to our knowledge, the first patient with a somatogonadal mosaicism in *COL5A1* responsible for the cEDS diagnosed in his son. Although the mosaicism was estimated with a VAF of 10–15% in blood, clinical examination did not reveal any signs of cEDS in the father.

In summary, our results show that half of MFS-affected individuals are sporadic, which is more frequent than usually expected. A significant proportion of these could be explained by parental post-zygotic mosaicism, which represents around 5% of probands with MFS. This first published estimation of parental mosaicism in MFS highlights the major impact for clinical practice of such mosaic variants and the importance of using analyses with enough sensitivity to detect them in our routine practice.

## Supplementary information

Supplementary Material 1

Supplementary Material 2
