# Supplementary material for: Parental mosaicism in Marfan and Ehlers–Danlos syndromes and related disorders
Source: Eur J Hum Genet. 2021 Jan 7;29(5):771–9. doi: 10.1038/s41431-020-00797-3 (PMC8110803; doi:10.1038/s41431-020-00797-3)
Supplement: Supplementary file 1 — Supplementary Material 1 [file 41431_2020_797_MOESM1_ESM.docx]

| Gènes | OMIM number | Transcript number |
| --- | --- | --- |
| *FBN1* | * 134797 | NM_000138.5 |
| *TGFBR1* | * 190181 | NM_004612.4 |
| *TGFBR2* | * 190182 | NM_003242.6 |
| *TGFB2* | * 190220 | NM_003238.6 |
| *ACTA2* | * 102620 | NM_001613.4 |
| *MYH11* | * 160745 | NM_002474.3 |
| *COL3A1* | * 120180 | NM_000090.3 |
| *MYLK* | * 600922 | NM_053025.4 |
| *SMAD3* | * 603109 | NM_005902.4 |
| *SLC2A10* | * 606145 | NM_030777.4 |
| *MFAP5* | * 601103 | NM_003480.4 |
| *TGFB3* | * 190230 | NM_003239.4 |
| *SKI* | * 164780 | NM_003036.4 |
| *PRKG1* | * 176894 | NM_006258.4 |
| *NOTCH1* | * 190198 | NM_017617.5 |
| *BGN* | * 301870 | NM_001711.6 |
| *EMILIN1* | * 130660 | NM_007046.4 |
| *FOXE3* | * 601094 | NM_012186.3 |
| *LOX* | * 153455 | NM_002317.7 |
| *MAT2A* | * 601468 | NM_005911.6 |
| *SMAD2* | * 601366 | NM_001135937.2 |
| *SMAD4* | * 600993 | NM_005359.6 |
| *COL1A2* | * 120160 | NM_000089.4 |
| *COL5A1* | * 120215 | NM_000093.5 |
| *COL5A2* | * 120190 | NM_000393.5 |
| *EFEMP2* | * 604633 | NM_016938.5 |
| *ELN* | * 130160 | NM_000501.4 |
| *FBN2* | * 612570 | NM_001999.4 |
| *FLNA* | * 300017 | NM_001456.3 |
| *ROBO4* | * 607528 | NM_019055.6 |
| *ADAMTSL4* | * 610113 | NM_019032.5 |
| *AEBP1* | * 602981 | NM_001129.5 |
| *LTBP2* | * 602091 | NM_000428.3 |
| *COL1A1* | * 120150 | NM_000088.3 |
| *PLOD1* | * 153454 | NM_000302.4 |

**Supplementary Material 1:** List of the 35 genes explored in the targeted Marfan syndrome and related disorders sequencing:
