# Supplementary material for: Parental mosaicism in Marfan and Ehlers–Danlos syndromes and related disorders
Source: Eur J Hum Genet. 2021 Jan 7;29(5):771–9. doi: 10.1038/s41431-020-00797-3 (PMC8110803; doi:10.1038/s41431-020-00797-3)

Supplementary Material 2: a. Craniofacial features of patient M0083 at 3 month of age (up) and 3 years of age (down). Note the premature aging facies, malar flattening, large and upfolded helix. b. Skin lesions of patient M1089 suggestive of Ehlers-Danlos syndrome classical type.

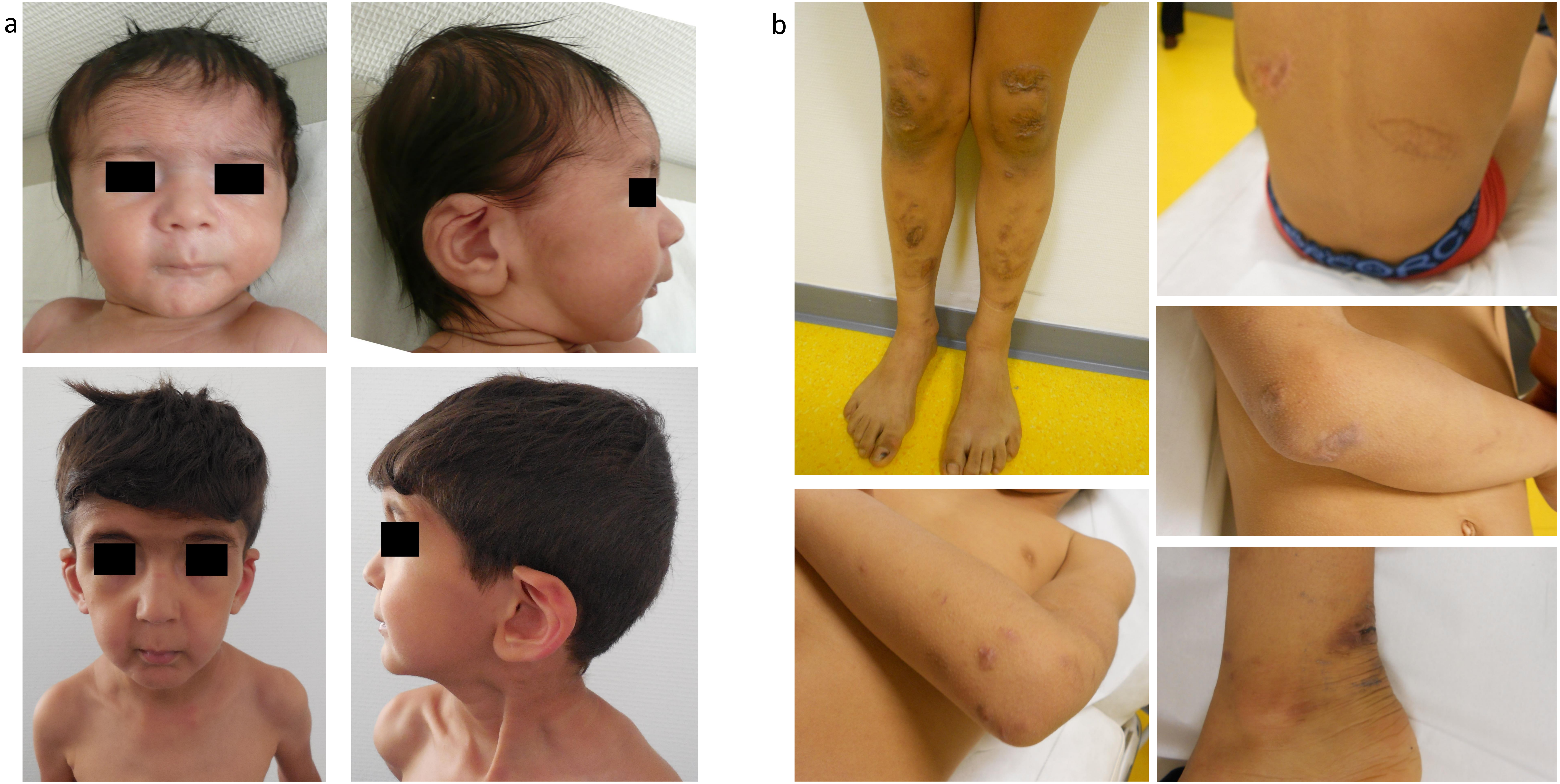

Supplement: Supplementary file 2 — Supplementary Material 2 [file 41431_2020_797_MOESM2_ESM.pdf]
